# Metagenomics reveals diversity and abundance of *meta*-cleavage pathways in microbial communities from soil highly contaminated with jet fuel under air-sparging bioremediation

**DOI:** 10.1111/j.1462-2920.2009.01943.x

**Published:** 2009-09

**Authors:** Maria V Brennerova, Jirina Josefiova, Vladimir Brenner, Dietmar H Pieper, Howard Junca

**Affiliations:** 1 Institute of Microbiology, Czech Academy of SciencesPrague, Czech Republic; 2 Department of Microbial Pathogenesis, HZI – Helmholtz Centre for Infection ResearchInhoffenstraße 7, D-38124 Braunschweig, Germany

## Abstract

The extradiol dioxygenase diversity of a site highly contaminated with aliphatic and aromatic hydrocarbons under air-sparging treatment was assessed by functional screening of a fosmid library in *Escherichia coli* with catechol as substrate. The 235 positive clones from inserts of DNA extracted from contaminated soil were equivalent to one extradiol dioxygenase-encoding gene per 3.6 Mb of DNA screened, indicating a strong selection for genes encoding this function. Three subfamilies were identified as being predominant, with 72, 55 and 43 fosmid inserts carrying genes, related to those encoding TbuE of *Ralstonia pickettii* PK01 (EXDO-D), IpbC of *Pseudomonas* sp. JR1 (EXDO-K2) or DbtC of *Burkholderia* sp. DBT1 (EXDO-Dbt), respectively, whereas genes encoding enzymes related to XylE of *Pseudomonas putida* mt-2 were not observed. Genes encoding oxygenases related to isopropylbenzene dioxygenases were usually colocalized with genes encoding EXDO-K2 dioxygenases. Functional analysis of representative proteins indicated a subcluster of EXDO-D proteins to show exceptional high affinity towards different catecholic substrates. Based on *V*_max_/*K*_m_ specificity constants, a task-sharing between different extradiol dioxygenases in the community of the contaminated site can be supposed, attaining a complementary and community-balanced catalytic power against diverse catecholic derivatives, as necessary for effective degradation of mixtures of aromatics.

## Introduction

Microbial communities are fundamental components of ecosystems, playing critical roles in the metabolism of organic matter. They are predominantly involved in detoxification of contaminated sites and organisms degrading a wide range of pollutants have been described. In nearly all situations, microbial reactions are the dominant processes driving natural attenuation or bioremediation processes.

Aromatic hydrocarbons such as benzene, toluene and ethylbenzene are common contaminants of soil and groundwater (Witzig *et al*., 2006) and are listed as priority pollutants by the US Environmental Protection Agency (http://www.epa.gov/waterscience/criteria/wqctable). Many microorganisms have evolved specialized pathways to use aromatic compounds as their sole carbon and energy sources (Harwood and Parales, 1996; Pieper, 2005). Metabolic pathways and encoding genes have been characterized in many bacterial strains, predominantly *Proteobacteria* and *Actinobacteria*. In the case of hydrophobic pollutants such as benzene, toluene, naphthalene, biphenyl or polycyclic aromatics, aerobic degradation is usually initiated by activation of the aromatic ring through oxygenation reactions catalysed by Rieske non-haem iron oxygenases (Gibson and Parales, 2000) or, as intensively described for toluene degradation, through members of the soluble diiron family of monooxygenases (Leahy *et al*., 2003).

While a relatively broad diversity of activation mechanisms is possible in aromatic degradation, these pathways usually converge in the formation of a few, usually dihydroxylated central intermediates, which are substrates for ring-cleavage dioxygenases. Methylsubstituted aromatics such as toluene or xylenes and aromatics containing two or more aromatic ring systems are usually degraded via the respective dihydroxylated derivatives and extradiol cleavage (Eltis and Bolin, 1996). Extradiol dioxygenases are thus key enzymes in the degradation of aromatic compounds and many of such proteins and their encoding sequences have been described, purified and characterized in the last decades. Examination of their relationships has shown that the extradiol dioxygenases can be categorized into three types belonging to different families. Type I enzymes belong to the vicinal oxygen chelate family (Eltis and Bolin, 1996), type II enzymes are related to LigB protocatechuate 4,5-dioxygenase (Sugimoto *et al*., 1999) and type III enzymes belong to the cupin superfamily (Dunwell *et al*., 2000). Importantly, novel extradiol dioxygenases are constantly being reported (Nogales *et al*., 2005; Moonen *et al*., 2008).

Most of our knowledge on how pollutants are degraded in the environment has been obtained by laboratory studies using isolated pollutant-degrading microorganisms or model communities. However, various culture-independent studies on genes coding for critical steps in aromatic degradation such as ring activation, or cleavage suggest that distinct and potentially numerically dominant sequence types of as-yet-unrecognized functional genes exist in natural and anthropogenically influenced environments and that the sequences from cultured strains are not likely to represent the microbial functional gene diversity in the environment (Yeates *et al*., 2000; Witzig *et al*., 2006). Most culture-independent surveys of catabolic gene diversity in contaminated environments rely on conserved nucleotide sequences used for the design of primers to survey the presence, abundance and diversity of catabolic genes encoding a defined group of enzymes supposed to be critical in the target environment. However, such definition of critical enzymes is often based on knowledge using isolates. As an example from culture-dependent studies, oxygenases of the benzene/toluene subfamily of Rieske non-haem iron oxygenases would be expected to be important in sites contaminated with benzene/toluene, whereas culture-independent studies covering a broad range of Rieske non-haem iron oxygenases discovered isopropylbenzene dioxygenases to be abundant in a respective contaminated site (Witzig *et al*., 2006).

One approach that relies neither on conserved nucleotide sequences nor on previous knowledge of isolates is the use of genomic libraries to retrieve genes catalysing a desired function from natural bacterial communities without cultivation. In fact, functional screening of metagenomic libraries has led to the discovery of novel genes encoding antibiotic resistance (Riesenfeld *et al*., 2004), ester- and glycosyl-hydrolases (Ferrer *et al*., 2005), polyphenol oxidase (Beloqui *et al*., 2006) and also given indications on the diversity of extradiol dioxygenases in coke plant wastewater (Suenaga *et al*., 2007).

The former army airbase Hradcany belongs to the region most intensively polluted with petroleum hydrocarbons in Northern Bohemia, Czech Republic. The contaminated area covers more than 28 ha with concentrations of total petroleum hydrocarbons ranging from 5000 to 55 000 mg kg^−1^ of dry soil. Significant amounts of aromatic hydrocarbons were detected and the concentration of BTEX (benzene, toluene, ethylbenzene, xylenes) was reaching 1000 mg kg^−1^ of dry soil (Machackova *et al*., 2008). Parts of the area have been under air-sparging treatment for 5 years and thus, the *in situ* stimulation of the natural microflora has presented us with an extraordinary opportunity to analyse the catabolic diversity for aromatic degradation that has been developed under real-site conditions.

In this study we used soil from the Hradcany area as a source for metagenomic DNA to characterize the diversity of genes encoding catechol 2,3-dioxygenases by a culture-independent function-based screening approach.

## Results

### Development and screening of metagenomic libraries

High-molecular-weight DNA was extracted from two soil samples representing both a highly polluted saturated zone in close vicinity to the water table (S) and an unsaturated zone with low level of contamination (W), which had bacterial densities culturable on R2A agar of 3.3 × 10^6^ and 6.3 × 10^5^ colony-forming units (cfu) per gram of dry soil. The metagenomic libraries were generated in *Escherichia coli* using pCCFOS fosmid vector and contained 8.7 × 10^4^ and 3 × 10^5^ clones respectively.

Both libraries were screened for catechol 2,3-dioxygenase activity by spraying with catechol. In the absence of inducer, yellow coloration due to the formation of 2-hydroxymuconic semialdehyde was not observed. However, 235 of 2.6 × 10^4^ clones from library S turned yellow on plates supplemented with l-arabinose, indicating that the high-copy amplification feature of the copy-controlled fosmid vector facilitated the visual detection of C23O expression. Whereas 0.91% of library S fosmid-containing *E. coli* strains exhibited C23O activity, only 19 of 3.5 × 10^4^ library W fosmid-containing *E. coli* strains (0.05%) were capable of cleaving catechol. This indicated C23O gene abundance to be correlated with the contamination level and a selective advantage of bacteria harbouring such function under the given environmental conditions.

Diversity and average insert size of the generated libraries were characterized by PFGE of NotI-digested plasmid DNA isolated from 15 randomly selected C23O-positive clones. Each of the NotI-digested fosmids showed, beside the expected 7.5 kb pCC1FOS vector band, a unique restriction fragment profile. The insert size of the analysed clones ranged from 23 to 42 kb and the approximate average insert length was 33 kb. It can thus be deduced that in library S one catechol 2,3-dioxygenase-encoding fosmid was recovered per 3.6 Mb of DNA screened, whereas in library W one catechol 2,3-dioxygenase-encoding fosmid was recovered per 61 Mb of DNA screened.

### PCR-based screening of all *meta*-activity selected metagenomic clones

New primers (Table 1) targeting eight main groups of extradiol dioxygenase genes (Fig. S1 in *Supporting information*) were used for PCR screening of fosmid clones exhibiting catechol 2,3-dioxygenase activity.

**Table 1:** Oligonucleotide primers used in this study.

Primers	Sequence (5′−3′)	Amplicon size (bp)
EXDO-A-F	ATGAAVAAAGGHGTWHTGCGHCCMGG	
EXDO-A-R1	GYGGCCADGCYTCNGGRTTG	430
EXDO-A-R2	ATRTCVAKVGADGTRTCGSTCATG	730
EXDO-B-F	TRACMGGHGTNHTGCGYCCVGGSCA	
EXDO-B-R	GCCRTGVCGSGTBGGVCCGAT	750
EXDO-C-F	CAYTAYCGYGACCGKATYGG	
EXDO-C-R1	TCRTCATGBGCYTTRTTGCTGCA	530
EXDO-C-R2	TCGTTSCGRTTDCCSGAVGGRTCGAAGAA	710
EXDO-D-F	AAYCCBGABCCNTGGCCNGA	
EXDO-D-R	GTYTSVCCNCGBGTVADVCCRTGRCG	380
EXDO-E-F	TAAAAGSWTGGGAYGAATACGA	
EXDO-E-R	TTTKGWKAAWAYATCCSCWGCTTT	575
EXDO-L-F	GACCAGGGSWTVGGYCACTA	
EXDO-L-R	TTRTGNCCCCAGATGCTGAT	430
EXDO-K1-F	TGGCGSATYGCYGTBCAGCMSGGCGA	
EXDO-K1-R	AGCATGAARTGRTGRATSCGYTTBGG	490
EXDO-K2-F	GAAAAAGTGGGTTTGATGGAGG	
EXDO-K2-R	CGCTTATGCCKCGTCATCACCC	810
bphAF668-3^a^	GTTCCGTGTAACTGGAARTWYGC	
bphAR1153-2^a^	CCAGTTCTCGCCRTCRTCYTGHTC	535
EXDO-Dbt-F	TCCGCATGGATTACAACC	
EXDO-Dbt-R	GATCTGTGGAACGGGCAA	423

a Primers have been described by Witzig and colleagues (2006).

Primers EXDO-A targeted C23O genes encoding subfamily I.2.A extradiol dioxygenases (examplified by *xylE* of *Pseudomonas putida* mt-2, V01161) primarily observed in γ-proteobacteria able to utilize monoaromatics. EXDO-B primers targeted catechol 2,3-dioxygenase-encoding genes related to LapB involved in alkylphenol degradation by *Pseudomonas* sp. strain KL28 (Jeong *et al*., 2003) whereas other primers targeted: catechol 2,3-dioxygenase-encoding genes of α-*Proteobacteria* related to XylE involved in *m-*xylene degradation by *Sphingomonas yanoikuyae* B1 (U23375; subfamily I.2.B, EXDO-C primers); catechol 2,3-dioxygenase-encoding genes of β- and γ-proteobacteria related to CbzE of *P. putida* GJ31 (AF19307) or TbuE of *Ralstonia pickettii* PK01 (U20258) involved in chlorobenzene or toluene degradation (subfamily I.2.C, EXDO-D primers): catechol 2,3-dioxygenase-encoding genes related to PheB of *Geobacillus thermoleovorans* A2 involved in phenol degradation (AF031325; EXDO-E primers), 2,3-dihydroxybiphenyl 1,2-dioxygenase-encoding genes of proteobacteria related to BphC of *Burkholderia xenovorans* LB400 involved in biphenyl degradation (CP000272; a subgroup of subfamily I.3.A, EXDO-K1 primers); 3-isopropylcatechol 2,3-dioxygenase-encoding genes of proteobacteria related to IpbC of *Pseudomonas* sp. strain JR1 involved in isopropylbenzene degradation (U53507; a second subgroup of subfamily I.3.A, EXDO-K2 primers); and catechol 2,3-dioxygenase-encoding genes of *Proteobacteria* related to TodE of *P. putida* F1 involved in toluene degradation (J04996; subfamily 1.3.B, EXDO-L primers).

Surprisingly, extradiol dioxygenases from the EXDO-A group, corresponding to the I.2.A subfamily (Eltis and Bolin, 1996), comprising the largest group of C23Os described by culture-dependent studies and assumed to be widely distributed in BTEX-contaminated environments, were not observed in the generated metagenomic libraries. In contrast, PCR analysis indicated the presence of two distinct groups of genes. When *E. coli* clones carrying fosmids exhibiting catechol 2,3-dioxygenase activity were used as template for a PCR screening with EXDO-D primers, specific amplification products of 380 bp were obtained from 72 of 235 library S fosmids (31%) and 1 of 19 library W fosmids. Screening with EXDO-K2 primers yielded specific amplification products of 824 bp in length for 55 library S-derived fosmids (23%).

Transposon mutagenesis was applied to inactivate the catechol 2,3-dioxygenase-encoding gene of fosmid s207, which exhibited a bright yellow coloration after being sprayed with catechol. Sequencing of the Tn*5*-insertion site revealed that the transposon has been inserted in a gene which exhibited 74% of sequence identity to *dbtC* of the dibenzothiophene degrading *Burkholderia* sp. strain DBT1 (Di Gregorio *et al*., 2004). Primers for the detection of *dbtC-*like genes (EXDO-Dbt) (Table 1) were thus constructed and applied for screening of the fosmid library. From a total of 43 fosmids of library S (18%) and one fosmid from library W an amplification product of the expected 423 bp size could be obtained, indicating EXDO-Dbt-encoding genes to be abundant in the soil under examination. Thus, 68% of all metagenomic clones were identified as carrying EXDO-encoding genes of the three major phylogenetic groups.

As the suggested EXDO-K2-encoding genes are usually part of catabolic operons associated with genes encoding Rieske non-haem iron oxygenases of the benzene/toluene/isopropylbenzene/biphenyl oxygenase branch (BTIB-RHDO), the previously designed primers bphA F668-3 and bphA R1153-2 targeting ISPα-encoding gene fragments of BTIB-RHDO (Witzig *et al*., 2006) were applied in an additional PCR-screening procedure. Specific 535 bp fragments could be amplified from 97 fosmids (40% of all selected library clones) and 45 of these were observed on metagenomic inserts, which also contained a EDDO-K2-encoding gene. Thus, 80% of all EXDO-K2-encoding metagenomic inserts also carried a BTIB-RHDO-encoding gene.

### Phylogenetic affiliation of catabolic genes

**Fig. 1:**
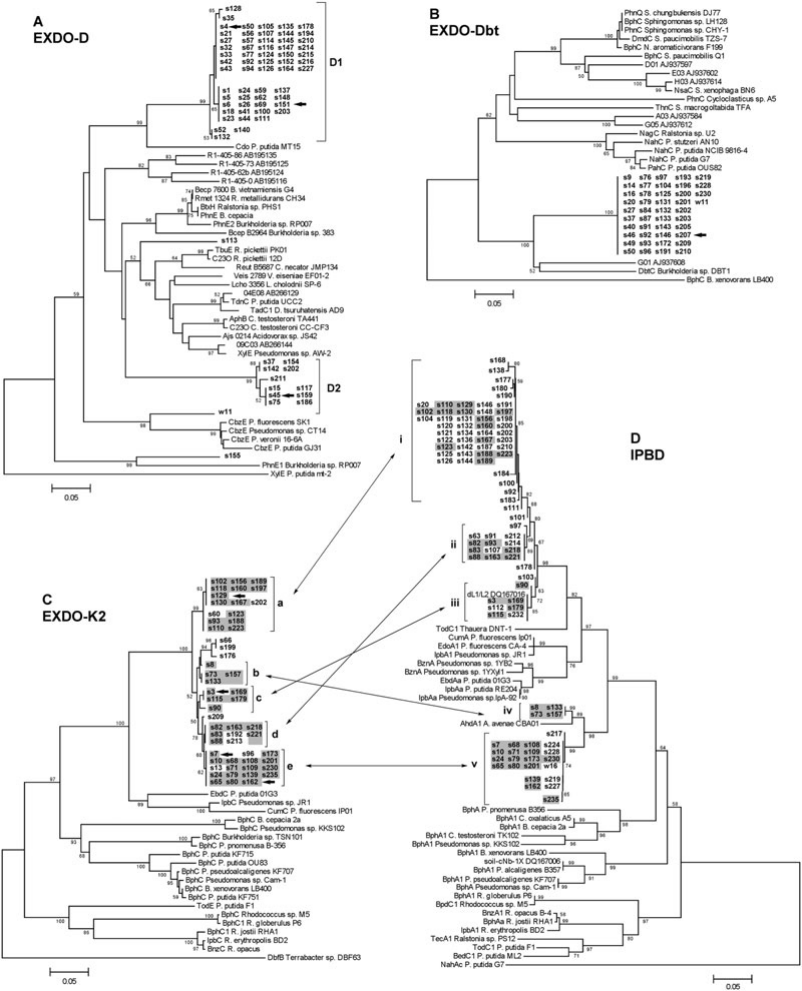
Evolutionary relationships of extradiol dioxygenases and of α-subunits of Rieske non-haem iron oxygenases found in phagemid metagenomic clones exhibiting catechol *meta*-cleavage activity. The evolutionary histories were inferred using the Neighbour-Joining method and the p-distance model. All positions containing alignment gaps and missing data were eliminated only in pairwise sequence comparisons. Phylogenetic analyses were conducted in MEGA4 using partial protein fragments. Bootstrap values above 50% from 100 neighbour-joining trees are indicated to the left of the nodes. The scale bar indicates amino acid differences per site. Proteins analysed biochemically in this study are indicated by arrows. A. Extradiol dioxygenases cluster D (EXDO-D). Sequences of closely related proteins from bacterial isolates or identified in culture independent studies (Kasuga *et al*., 2007; Suenaga *et al*., 2007) were included in the analysis and XylE of *P. putida* mt-2 is shown as an out-group. B. Extradiol dioxygenases related to DbtC (EXDO-Dbt). Sequences of closely related proteins from bacterial isolates or identified in culture independent studies (Sipila *et al*., 2006) were included in the analysis and BphC of *B. xenovorans* LB400 is shown as an out-group. C. Extradiol dioxygenases cluster K2 (EXDO-K2). Sequences of closely related proteins from bacterial isolates were included in the analysis and DbfB of *Terrabacter* sp. DBF63 is shown as an out-group. Proteins that were encoded on a metagenomic insert, which also encodes an isopropylbenzene dioxygenase are shaded in grey. Colocalization of EXDO-K2-encoding genes and of isopropylbenzene dioxygenase (IPBD)-encoding genes is indicated by arrows. D. α-Subunits of Rieske non-haem iron oxygenases related to IPBD. Sequences of closely related proteins from bacterial isolates or identified in culture independent studies (Witzig *et al*., 2006) were included in the analysis and NahAc of *P. putida* G7 is shown as an out-group. Proteins that were encoded on a metagenomic insert, which also encodes an EXDO-K2, are shaded in grey.

Sequence analysis of the obtained fragments showed that all products were due to amplification of fragments of the expected catabolic gene groups. In case of EXDO-D-encoding genes, two distinct clusters of genes were observed with similarity between members of the different clusters of 62–67%. The 15 gene polymorphisms of cluster D1, comprising 59 clones, encoded four peptide variants differing by one to two amino acids in the 113- to 114-amino-acid sequence analysed (Fig. 1A). The highest similarity (79–81% of identity) was observed with CdoII extradiol dioxygenase of *P. putida* MT15 (Keil *et al*., 1985). A subgroup of 11 gene fragments (Fig. 1A, cluster D2) encoded three distinct peptides differing by up to three amino acids, which showed similarity to a cluster of proteins comprising, among others, BtxH of *Ralstonia* sp. PSH1 (DQ834383; 68–71% identity) and to a cluster of proteins comprising, among others, TbuE of *R. pickettii* PK01 (63–71% identity) (Kukor and Olsen, 1991). The deduced protein sequence of clone s113 was most closely related to the previously mentioned proteins (75–83% sequence identity), whereas the deduced protein sequence of clone s115 resembled that of PhnE1 of *Burkholderia* sp. RP007 (Laurie and LloydJones, 1999) and the deduced protein sequence of clone w11 sharing 78–82% similarity with CbzE chlorocatechol 1,2-dioxygenases of various *Pseudomonas* strains (Gobel *et al*., 2004).

The 55 EXDO-K2-encoding genes could be differentiated into 14 phylotypes sharing > 96% of sequence identity and encoded 11 different peptides. The deduced peptide sequences (Fig. 1C) showed the highest similarity (78–81% identity) to extradiol dioxygenases of gene clusters reported to be involved in the degradation of isopropylbenzene such as IpbC of *Pseudomonas* sp. JR1 (Pflugmacher *et al*., 1996) or ethylbenzene such as EbdC of *P. putida* 01G3 (Chablain *et al*., 2001).

The third group of 44 gene fragments (43 from library S and one from library W) amplified by EXDO-Dbt primers were identical in sequence and encoded peptides showing similarity to extradiol dioxygenases involved in the degradation of naphthalene (see Fig. 1B) (55–64% sequence identity) and specifically to DbtC of *Burkholderia* sp. DBT1 (Di Gregorio *et al*., 2004).

All gene fragments amplified by the bphA F668-3 and bphA R1153-2 primers encoded peptides, which in a phylogenetic analysis were closely related to a cluster of proteins comprising, among others, isopropylbenzene dioxygenases from *Pseudomonas fluorescens* IP01, *Pseudomonas* sp. JR1 and *P. putida* RE204 (Eaton and Timmis, 1986; Habe *et al*., 1996; Pflugmacher *et al*., 1996). The deduced protein sequences [herein termed isopropylbenzene dioxygenase (IPBD)], could be grouped into two distinct clusters with sequence identities between cluster members of 73–75% (see Fig. 1D). The 71 clones defining the first cluster (comprising groups i, ii and iii in Fig. 1D, all derived from library S) showed 16 gene polymorphisms, encoding eight peptide variants differing by up to 10 amino acids in the 160- to 161-amino-acid sequence analysed (Fig. 1D). The highest similarity (84–91% identity) was observed with TodC1 of *Thauera* sp. strain DNT-1 (Shinoda *et al*., 2004) and various enzymes termed isopropylbenzene dioxygenases. Twenty-six gene fragments (Fig. 1D, groups iv and v, 25 fragments derived from library S and one from library W) encoding two distinct peptides (encoded by four gene polymorphisms) differing by seven amino acids showed the highest similarity (91–95%) with AhdA1 of the chlorobenzene degrader *Acidovorax avenae* CBA1 (Monferran *et al*., 2005).

### Substrate specificity of extradiol dioxygenases

Selected clones representing the major EXDO phylogenetic groups identified above, with clones s7 and s162 containing enzymes identical in the sequence stretch analysed, were selected for kinetic characterization. Besides 3-methylcatechol, catechol, 2,3-dihydroxybiphenyl and 1,2-dihydroxynaphthalene were used as substrates as they represent the preferred substrates of enzymes closely related to those identified in the current study. All activities were partially purified by anionic exchange chromatography before kinetic characterization to exclude that the activity was due to coexpression of different extradiol dioxygenases.

Both EXDO-K2_s162 and EXDO-K2_s7 showed nearly identical substrate spectra (Table 2), but pronounced differences in expression level, which was expected and most probably to their different localization on the metagenomic insert. Both dioxygenases showed the highest substrate preference, expressed by the relative specificity constant *V*_max_/*K*_m_, for 2,3-dihydroxybiphenyl followed by 3-methylcatechol. Catechol was less preferred; however, both the *K*_m_ and *V*_max_ values differed from those measured with 2,3-dihydroxybiphenyl only by a factor of up to 6, contrasting 2,3-dihydroxybiphenyl 2,3-dioxygenases such as the enzymes from *B. xenovorans* LB400 or *Rhodococcus globerulus* P6, where differences in substrate preference by two to five orders of magnitude were observed, specifically due to high *K*_m_ values with catechol in the mM range (Vaillancourt *et al*., 2002; McKay *et al*., 2003). Thus, both enzymes can be regarded as promiscuous, also compared with enzymes like TlpE where also two orders in magnitude between *V*_max_/*K*_m_ values for 2,3-dihydroxybiphenyl and catechol could be observed (Beil *et al*., 1999). Kinetic properties similar to those of EXDO-K2_s162 and EXDO-K2_s7 were also observed for EXDO-K2_s3 and EXDO-K2_s129 (Table 2).

**Table 2:** Substrate specificity of partially purified catechol 2,3-dioxygenases.

Clone	Substrate	Activity in extract^a^ (U per gram of protein)	*K*_m_ (μM)^b^	*V*_max_^c^	*V*_max_/*K*_m_^d^
s7	Catechol	17 ± 3	5.3 ± 0.7	17	0.15
	3-Methylcatechol	95 ± 8	4.6 ± 0.4	100	1
	2,3-Dihydroxybiphenyl	42 ± 5	0.84 ± 0.08	41	2.25
s162	Catechol	90 ± 10	4.7 ± 0.4	18	0.16
	3-Methylcatechol	510 ± 35	4.3 ± 0.7	100	1
	2,3-Dihydroxybiphenyl	175 ± 15	1.0 ± 0.10	47	2.02
S3	Catechol	41 ± 5	8.3 ± 1.1	23	0.12
	3-Methylcatechol	180 ± 15	4.5 ± 0.6	100	1
	2,3-Dihydroxybiphenyl	56 ± 6	2.1 ± 0.2	30	0.64
S129	Catechol	40 ± 6	6.7 ± 0.6	8	0.10
	3-Methylcatechol	520 ± 35	9.3 ± 1.1	100	1
	2,3-Dihydroxybiphenyl	205 ± 20	2.1 ± 0.2	36	1.59
S4	Catechol	955 ± 85	1.4 ± 0.2	49	0.63
	3-Methylcatechol	2890 ± 190	1.8 ± 0.3	100	1
	2,3-Dihydroxybiphenyl	70 ± 8	3.7 ± 0.6	4	0.02
s151	Catechol	25 ± 5	1.1 ± 0.2	66	1.9
	3-Methylcatechol	42 ± 7	3.5 ± 1.5	100	1
	2,3-Dihydroxybiphenyl	3 ± 1	3.0 ± 0.5	6	0.07
s45	Catechol	7510 ± 410	0.27 ± 0.03	193	1.93
	3-Methylcatechol	3730 ± 220	0.27 ± 0.10	100	1
	2,3-Dihydroxybiphenyl	560 ± 30	0.61 ± 0.08	15	0.064
s207	Catechol	30 ± 5 (6.6 ± 0.7)	35 ± 5	12	0.046
	3-Methylcatechol	385 ± 30	13.6 ± 1.9	100	1
	2,3-Dihydroxybiphenyl	160 ± 15	4.2 ± 0.7	26	0.84
	1,2-Dihydroxynaphthalene	450 ± 35	11.6 ± 1.4	668	7.84

a Activities (U per gram protein) were determined at 100 μM substrate concentrations and at pH values of 8.0, except for the activity with 1,2-dihydroxynaphthalene, which was determined at a pH of 5.5. Activity with catechol at pH 5.5 is indicated in parentheses.

b *K*_m_ values were determined in partially purified enzyme fractions.

c *V*_max_ is given in percentage of the activity with 3-methylcatechol. Activities were determined in partially purified enzyme fractions. In case of catechol 2,3-dioxygenase s207, relative activities were corrected for the fact that at pH 5.5 the enzyme exhibits only 22% of the activity determined at pH 8.0.

d *V*_max_/*K*_m_ values were calculated relative to the value for 3-methylcatechol set as 1.

Catechol was the preferred substrate for both EXDO-D_s45 and EXDO-D_s151, with the two orders of magnitude difference in preference being due mainly to a significant lower *V*_max_ of the enzymes with 2,3-dihydroxybiphenyl compared with catechol. Both enzymes differed significantly in their affinity towards the substrates, with EXDO-D_s45 exhibiting *K*_m_ values usually lower than 1 μM, even with 2,3-dihydroxybiphenyl. Similar low *K*_m_ values with catechol and 3-methylcatechol were previously observed for TbuE extradiol dioxygenase of *R. pickettii* PK01 (Kukor and Olsen, 1996). However, no further information on the affinity of related enzymes for their substrates is available. EXDO-D_s4 showed kinetic properties similar to EXDO-D_s45; however, a higher relative activity with 3-methylcatechol was evident (Table 2).

Of the enzymes tested, only EXDO-Dbt_s207 exhibited activity with 1,2-dihydroxynaphthalene above the detection limit. Unfortunately, information on such activity is scarce due to the high autooxidation rate of the substrate (Kuhm *et al*., 1991a). However, some enzymes belonging to the group of 1,2-dihydroxynaphthalene dioxygenases (see Fig. 1B) have been characterized. Even though enzymes such as ThnC of *Sphingopyxis macrogoltabida*, BphC of *Sphingomonas paucimobilis* Q1 or NsaC of *Sphingomonas xenophaga* BN6 show high activity with 2,3-dihydroxybiphenyl and methylcatechols, they usually exhibited higher activity with 1,2-dihydroxynaphtahlene as a substrate, and even catechol was shown to be transformed with reasonable activity (Kuhm *et al*., 1991a,b; Andujar and Santero, 2003). In addition, members of this group also transform 5,6-dihydroxytetralin (Andujar *et al*., 2000) and are involved in the degradation of dibenzothiophen via 1,2-dihydroxydibenzothiophen (Di Gregorio *et al*., 2004). Although 1,2-dihydroxynaphthalene was the preferred substrate for EXDO-Dbt_s207, both 2,3-dihydroxybiphenyl and 3-methylcatechol were transformed with reasonable activity. As even catechol was transformed, EXDO-Dbt_s207 can be regarded to be of broad substrate specificity.

## Discussion

In the current study the diversity of extradiol dioxygenases at a contaminated site under bioremediation treatment was analysed, allowing the characterization of gene polymorphisms which have been selected under stringent environmental conditions. Such culture-independent characterization of predominant activities is a major goal in identifying important catabolic routes under *in situ* conditions.

Culture-dependent studies have indicated a broad diversity of catabolic genes expressing extradiol dioxygenase activity. Catechol 2,3-dioxygenases (family I.2) (Eltis and Bolin, 1996) and LigB-type 2,3-dihydroxyphenylpropionate dioxygenases (Diaz *et al*., 2001) are the dominant groups exhibiting significant activity against catechol, but also TodE-like extradiol dioxygenases, which belong to subfamily I.3.B, exhibit such activity (Beil *et al*., 1999). Based on culture-dependent studies, the *xylE* gene encoding EXDO-A catechol 2,3-dioxygenase of *P. putida* mt2 as well as related enzymes and their encoding genes are classically used as a marker for extradiol dioxygenase activity including characterization of the catabolic potential of contaminated sites (Margesin *et al*., 2003). In fact, respective genes have been reported in various catabolic operons, and have recently also been observed as being predominant at a site mainly contaminated with benzene (Junca *et al*., 2004). However, for the downstream processing of extradiol ring-cleavage products, two different catabolic branches have been described. The degradation of benzene via extradiol cleavage of catechol necessitates the presence of the dehydrogenase branch of the *meta*-cleavage pathway suited for metabolism and specifically rapid dehydrogenation of the ring-cleavage product 2-hydroxymuconic semialdehyde (Harayama *et al*., 1987). Respective genes are typically present in operons comprising a EXDO-A-encoding gene, but typically absent from operons involved in the degradation of toluene, isopropylbenzene or biphenyl (Eaton and Timmis, 1986; Hofer *et al*., 1994) indicating that the last type of operons is not suited for benzene degradation. In contrast, the degradation of toluene, isopropylbenzene or biphenyl necessitates the presence of the hydrolytic branch of the *meta*-cleavage pathway for rapid hydrolysis of the ketonic ring-cleavage products (Harayama *et al*., 1987). As the hydrolytic branch is typically comprised together with a BTIB-RHDO in operons encoding a subfamily I.3.A or I.3.B extradiol dioxygenase (Eaton and Timmis, 1986; Hofer *et al*., 1994), it can be speculated that contamination with toluene/isopropylbenzene/biphenyl would select for subfamily I.3 extradiol dioxygenase comprising pathways. In fact, genes encoding a subgroup of extradiol dioxygenases of subfamily I.3 most closely related to isopropylcatechol 2,3-dioxygenases termed EXDO-K2 were observed to be abundant at the site under study.

EXDO-K2-encoding genes in isolates reported thus far are localized in an operon with genes encoding an IPBD. In fact, 45 of 55 clones expressing EXDO-K2 also showed the presence of such a gene, with the frequency of co-occurrence being as expected for genes being separated by 5.5 kb on 33 kb metagenomic inserts. A comparison of the phylogenies of respective EXDO-K2- and IPBD-encoding gene fragments (Fig. 1C and D) indicated that both segments had co-evolved and support the notion that they are localized in the same gene cluster. However, while EXDO-K2-encoding genes were obviously generally clustered with IPBD-encoding genes, an overall of 52 IPBD-encoding genes (51 from library S and one from library W) were observed on metagenomic inserts, where no EXDO-K2-encoding gene was localized and specifically genes of subgroup i (Fig. 1D) were often not clustered with EXDO-K2-encoding genes. Some IPBD-encoding genes were localized on inserts which showed the presence of an EXDO-D- or EXDO-Dbt-encoding gene and 23 on inserts which showed none of the above-mentioned EXDO-encoding genes. As all of those inserts express EXDO activity, it may be speculated that IPBD-encoding genes cluster with thus far unknown extradiol dioxygenases. Overall, these results indicate that IPBD-encoding genes have different genetic environments and may be recruited for different metabolic pathways. It is interesting to note that IPBD gene fragments were also observed to be abundant in the benzene-contaminated site mentioned above (Witzig *et al*., 2006); however, respective isolates did not show the presence of an EXDO-K2, but harboured an EXDO-A.

To date, a few reports are available to assess the diversity of catechol 2,3-dioxygenases beyond EXDO-A proteins. Sipila and colleagues (2006) targeted extradiol dioxygenases of the subfamily I.3.E, comprising among others NahC from *Pseudomonas* G7 and observed sequence types similar to EXDO-Dbt reported here to be predominant in an artificially PAH-polluted birch rhizosphere associated soil. Kasuga and colleagues (2007) analysed the diversity of catechol 2,3-dioxygenases based on primers designed on a limited but diverse set of catechol 2,3-dioxygenase-encoding genes, including *cdo* of *P. putida* MT15 (Sei *et al*., 1999) in unpolluted environments. Among others, EXDO-D variants could be shown to be abundant in three river water samples (Kasuga *et al*., 2007). Finally, Suenaga and colleagues (2007) analysed extradiol dioxygenases diversity in activated sludge used to treat coke plant wastewater by metagenomic library screening. In the library analysed, the most abundant sequence type showed high similarity to enzymes exemplified by BphC of *Bacillus* sp. JF8 (Hatta *et al*., 2003) or DbfB of *Paenibacillus* sp. YK5 (Iida *et al*., 2006). However, sequences similar to EXDO-D were also observed to be abundant in that study whereas sequences indicating the presence of EXDO-A proteins were practically absent. It thus seems that EXDO-A proteins are selected in the environment only under exceptional conditions. The environmental importance of EXDO-D proteins, in contrast, has been discussed in only a few cases. Most importantly, EXDO-D type sequences were observed in microorganisms mineralizing chloroaromatics via the *meta*-cleavage pathway (Gobel *et al*., 2004). However, the capability to efficiently transform 3-chlorocatechol is so far limited to a small subcluster of EXDO-D proteins. It had also been proposed that EXDO-D proteins may be adapted to function in hypoxic environments (Kukor and Olsen, 1996). Kinetic analysis of selected variants indicates that enzymes of subcluster EXDO-D2 are, like TbuE of *R. pickettii* PK01, of high affinity for their catecholic substrate, possibly giving strains harbouring such activities a selective advantage. However, such high affinity is not a general feature of EXDO-D-type dioxygenases, and enzymes of cluster EXDO-D1 are functionally resembling, regarding their affinity, typical EXDO-A proteins (Junca *et al*., 2004). Overall, it is interesting to note that all enzymes analysed here are on the one hand characterized by certain level of promiscuity, but on the other hand, a task sharing between the different variants can be proposed, having 2,3-dihydroxybiphenyl, 1,2-dihydroxynaphthalene and catechol as preferred substrates.

The majority of the fosmid clones of library S exhibiting extradiol dioxygenase contained at least one EXDO gene of one of the three subfamilies described above and thus a member of a previously described subfamily. It remains to be established if a similar situation holds for the remaining extradiol dioxygenase-expressing fosmids. Similarly, the screening by Suenaga and colleagues (2007), when taking into account the immense work performed on extradiol dioxygenases since the systematics of extradiol dioxygenases reported by Eltis and Bolin (1996), typically identified enzymes similar to those previously reported. This clearly shows that at least in case of extradiol dioxygenases, the knowledge gained by cultured isolates is covering activities important for aromatic metabolism in the field.

The current work also underlines the *in situ* relevance of extradiol dioxygenases in contaminated environments. To our knowledge, no other function has so far been reported to be encoded at densities of one gene copy per bacterial genome equivalent over the whole bacterial community metagenomic DNA of an environmental sample. This exceptionally high extradiol dioxygenase density in the highly polluted saturated zone indicates that this function confers a positive biological fitness to the microbial community members hosting it. Taking into account that the microbial community of the site under study comprises at least members of 14 bacterial orders (Kabelitz *et al*., 2009) the encoding genes might be concentrated in multiple copies in specific bacterial hosts.

## Experimental procedures

### Soil samples

Two soil samples were collected at different depths of the former army airbase Hradcany, a site being under air-sparging treatment for 5 years (Machackova *et al*., 2008). Sample ‘S’ was taken from the saturated zone at a depth of 5.3 m, where pollution by total petrol hydrocarbons remained at 22 000 mg kg^−1^ of dry soil, comprising 22 mg kg^−1^ of benzene, 23 mg kg^−1^ of toluene, 410 mg kg^−1^ of ethylbenzene and 500 mg kg^−1^ of xylenes. Analyses of the ground water have shown that in addition to BTEX (6600 μg l^−1^) polycylic aromatic hydrocarbons (PAH), such as naphthalene (21 μg l^−1^), phenanthrene (1.9 μg l^−1^), fluoranthene (0.24 μg l^−1^), pyrene (0.22 μg l^−1^), chrysene (0.15 μg l^−1^) and benzanthracene (0.10 μg l^−1^), are also present. Sample ‘W’ was collected from the willow rhizosphere at 2.5 m depth where the concentration of total petrol hydrocarbons (TPH) was only 60 mg kg^−1^ of dry soil with the concentration of BTEX < 0.5 mg kg^−1^. Both samples consisted of sand particles with medium and fine grain size (83.7% of dry soil) and a minor fraction of clay particles (1.67%). Water suspensions (1:1) of S and W soil were showing pH of 6.25 and 6.9 respectively. *In situ* monitoring of groundwater indicated a redox potential of 60 mV, dissolved oxygen of 1.6 mg l^−1^ and a pH of 6.4.

### DNA extraction and library construction

High-molecular-weight soil DNA was essentially recovered as described previously (Selenska-Pobell *et al*., 2001). In brief, bulk soil and soil loosely attached to willow tree roots were separately homogenized and sieved to remove plant debris and particles > 3 mm. These soil samples were analysed for colony-forming units by suspending 1 g of soil in 9 ml of 0.9% NaCl, agitating for 1 h at 4°C followed by plating of appropriate dilutions on R2A agar plates.

Ten grams of each soil sample were suspended in 30 ml of lysis buffer (0.12 M Na_2_HPO_4_, 0.02% Tween 20, 10% SDS; pH 8), agitated gently (30 min, 70°C), and then RNA and proteins were removed by addition of RNase A (20 μg ml^−1^, 30 min at 37°C) and subsequently of proteinase K (100 μg ml^−1^, 30 min at 65°C). Soil particles were removed by centrifugation (15 min, 8000 *g*, 10°C), the supernatant was kept on ice (1.5 h) and precipitates were removed by centrifugation (30 min, 15 000 *g*, 4°C). Nucleic acids were precipitated by addition of 0.1 volumes of 50% PEG 6000 and 0.4 volumes of 5 M NaCl. After incubation overnight at 4°C, precipitated nucleic acids were collected by centrifugation (30 min, 15 000 *g*, 4°C), re-suspended in 5 ml of TE buffer (pH 8) and purified using NucleoBond AXG 100 cartridges (Macherey-Nagel).

Metagenomic DNA was blunt-ended and 2.5 μg was subjected to pulsed-field gel electrophoresis (PFGE) in SeaKem® Gold Agarose (Cambrex Bio Science Rockland) using a CHEF-DR III system (Bio-Rad Laboratories), at 1–10 s switch, 6 V cm^−1^, 120° fixed angle and 12 h run time. Linearized 36 kb fosmid and 8–48 kb CHEF DNA size standard (Bio-Rad Laboratories) were used as markers. The bulk of metagenomic DNA was represented by fragments with sizes of 10–50 kb. The gel area containing DNA fragments of 25–45 kb was excised, embedded into 1.2% Certified Low-Melt agarose (Bio-Rad Laboratories) and subjected to a second PFGE for 3 h. DNA was extracted by GELase treatment and subjected to ethanol precipitation. End-repaired DNA was cloned into the fosmid copy-control pCC1FOS™ vector (Epicentre®). Ligation, packaging into phage particles and transfection in *E. coli* EPI300-T1^R^ was performed according to the recommended protocol (Epicentre®). The primary libraries were stored transfected into *E. coli* EPI300-T1^R^.

### Selection of fosmid clones exhibiting catechol 2,3-dioxygenase (C23O) activity

*Escherichia coli* EPI300-T1^R^ harbouring fosmid clones were plated at a density of approximately 300 colonies on LB agar plates, supplemented with 25 μg ml^−1^ chloramphenicol and 0.01% l-arabinose as an inducer of plasmid amplification. Plates were incubated at 37°C and sprayed with filter-sterilized catechol (1%) after 36 h of incubation. Colonies that turned yellow due to extradiol cleavage of catechol were purified on LB agar plates and stored as glycerol cultures at −80°C.

### PCR screening of activity-selected clones

PCR-based screening was performed with primer sets (Table 1) targeting genes from eight different extradiol dioxygenases type I subfamilies (see *Supporting information*), with a primer set targeting *dbtC* genes encoding extradiol dioxygenases involved in dibenzothiophen degradation (Table 1) and with the primer set bphAf668-3/bphAr1153-2 (Witzig *et al*., 2006) for amplification of Rieske non-haem iron oxygenase ISPa gene segments of the toluene/biphenyl subfamily. Amplification with EXDO primers A, C, D, E, L, K1 and K2 was performed by touchdown PCR with six touchdown cycles, starting at an annealing temperature of 61°C followed by a decrease in annealing temperature of 1°C per cycle and 25 PCR cycles at a constant annealing temperature of 55°C. Amplification with EXDO-B primers was carried out by 30 PCR cycles at an annealing temperature of 62°C and amplification using the dbtCf/dbtCr primers by 32 PCR cycles at an annealing temperature of 58°C. PCR products were separated on 1.5% agarose gels and visualized by ethidium bromide staining.

### General DNA manipulations

Plasmid preparation, restriction endonuclease digestion, DNA ligation, agarose gel electrophoresis and other standard recombinant DNA techniques were carried out as described by Sambrook and colleagues (1989). Restriction-fragment length analysis of selected fosmid clones was performed after digestion with NotI and pulse-field gel electrophoresis for 12 h as described above.

The EZ-Tn5™ <T7/KAN-2> Promoter Insertion Kit (Epicentre®) was applied for Tn*5*-based targeting of catabolic gene clusters. Fosmid DNA of clones, encoding catechol extradiol dioxygenase activity, was isolated and 250 ng aliquots were used for *in vitro* transposon insertion reaction with subsequent electroporation into TransforMax™ EC100-T1^R^ electrocompetent *E. coli.* Transposon insertion clones were grown on LB plates containing 50 μg ml^−1^ kanamycin and sprayed with catechol (1%). Clones in which catechol 2,3-dioxygenase activity was absent were used for sequence analysis of the transposon insertion sites.

### DNA sequencing and phylogenetic analysis

For sequencing ends of fosmid clones and transposon insertion sites, fosmid DNA was isolated at conditions inducing high-copy amplification and purified with the QIAGEN Plasmid Mini Kit system (QIAGEN, Hilden, Germany). Primers used for sequencing reations were the pCC1™ forward and reverse sequencing primers (Epicentre®) and the transposon-specific primers KAN-2 FP-1 and KAN-2 RP-1 (Epicentre®). Sequencing of catechol 2,3-dioxygenase gene fragments was performed on PCR products purified with QIAquick PCR Purification Kit with primers used for sequencing reactions being the same as those used in the original PCR.

Sequencing was performed with an ABI PRISM BigDye Terminator v1.1 Ready Reaction Cycle Sequencing Kit (Applied Biosystems, Foster City, CA) and an ABI PRISM 3100 Genetic Analyser (Applied Biosystems). Raw sequence chromatograms from both strands were assembled with Sequencher software version 4.0.5 (Gene Codes Corporation). Assembled contigs were used for DNA and protein similarity searches on GenBank databases performed with blastn and blastp programs of the National Center for Biotechnology Information website (http://ncbi.nlm.nih.gov). Translated protein sequences were aligned with clustalx 1.83 using default values. Phylogenetic trees were constructed with MEGA4 (Tamura *et al*., 2007) using the Neighbour-Joining (N-J) algorithm (Saitou and Nei, 1987) with p-distance correction and pairwise deletion of gaps and missing data. A total of 100 bootstrap replications were performed to test for branch robustness.

The sequences of genes and PCR fragments reported in this study are available under the EMBL/GenBank/DBBJ Accession No. EU555067 to EU555117 and EU884652 to EU884919.

### Enzyme purification and sequencing

*Escherichia coli* EPI300-T1R-expressing C23O genes were grown at 37°C in Luria broth medium containing 0.01% l-arabinose and 25 μg ml^−1^ chloramphenicol and then harvested during late exponential growth. Preparation of cell extracts, partial purification of catechol 2,3-dioxygenases through anionic exchange chromatography, SDS-polyacrylamide gel electrophoresis (SDS-PAGE) and N-terminal amino acid sequencing were carried out as previously described (Junca *et al*., 2004).

### Enzyme assays

Extradiol dioxygenase activities were recorded on a UV 2100 spectrophotometer (Shimadzu Corporation) in 50 mM air-saturated K/Na-phosphate (pH 8.0) with catechol, 3-methylcatechol or 2,3-dihydroxybiphenyl as substrates, using extinction coefficients of reaction products previously described (catechol, ε_375_ = 36 000 M^−1^ cm^−1^; 3-methylcatechol, ε_388_ = 13 600 M^−1^ cm^−1^; 2,3-dihydroxybiphenyl, ε_434_ = 21 700 M^−1^ cm^−1^) (Hirose *et al*., 1994; Heiss *et al*., 1995). Activity with 1,2-dihydroxynaphthalene (0.1 mM supplied from a 50 mM stock solution in tetrahydrofuran) was determined in 50 mM acetic acid/NaOH (pH 5.5) using a reaction coefficient of ε_331_ = 2600 M^−1^ cm^−1^ (Kuhm *et al*., 1991a). In order to compare activities with different substrates, the activity with catechol was also analysed at pH 5.5 (catechol, ε_375_ = 16 400 M^−1^ cm^−1^).

Protein concentrations were determined by the method of Bradford (1976) with bovine serum albumin as the standard. One unit of enzyme activity was defined as the amount of enzyme that forms 1 μmol product per minute. Specific activities are expressed as units per gram of protein.

*V*_max_ and *K*_m_ values were determined using 1–100 μM substrate in air-saturated buffer, and kinetic data were calculated from the initial velocities using the Michaelis–Menten equation by non-linear regression (KaleidaGraph, Synergy Software).

## Supporting information

**Fig. S1.** Dendrogram of 196 members of the extradiol dioxygenase type 1 family, representing the main evolutionary branches. The labelled groups compressed in triangles are the branches selected as targets for amplification. The corresponding CDS were collected and used for the primer design resulting in the primer set reported in Table 1.
