# Supplementary material for: Metagenomics reveals diversity and abundance of meta-cleavage pathways in microbial communities from soil highly contaminated with jet fuel under air-sparging bioremediation
Source: Environ Microbiol. 2009 Sep;11(9):2216–27. doi: 10.1111/j.1462-2920.2009.01943.x (PMC2784041; doi:10.1111/j.1462-2920.2009.01943.x)
Supplement: Supplementary file 1 [file emi0011-2216-SD1.doc]

### Figure SP1


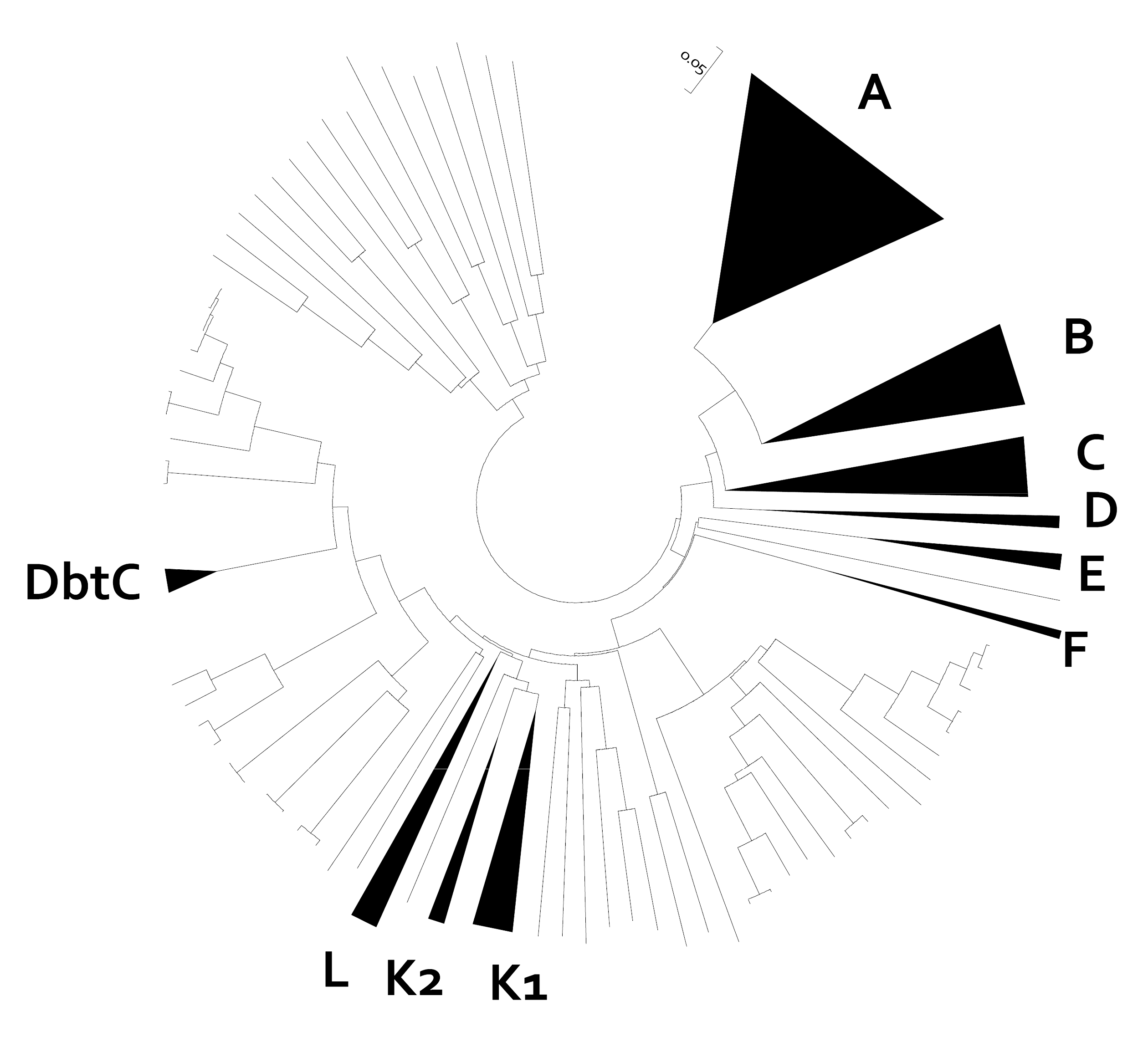


Dendrogram of 196 members of the extradiol dioxygenase type 1 family, representing the main evolutionary branches. The labeled groups compressed in triangles are the branches selected as targets for amplification. The corresponding CDS were collected and used for the primer design resulting in the primer set reported in Table 1.
